# Drought-tolerant rice, weather index insurance, and comprehensive risk management for smallholders: evidence from a multi-year field experiment in India

**DOI:** 10.1111/1467-8489.12342

**Published:** 2019-10-15

**Authors:** Patrick S. Ward, Simrin Makhija, David J. Spielman

**Affiliations:** † Patrick S. Ward is Assistant Professor of Environmental Economics and Policy at the Duke Kunshan University, Kunshan, Jiangsu Province, China. Patrick S. Ward is also former Research Fellow at the International Food Policy Research Institute, Washington, District of Columbia, USA. Simrin Makhija is Senior Research Analyst and David J. Spielman is Senior Research Fellow with the International Food Policy Research Institute, Washington, District of Columbia, USA

**Keywords:** agriculture, drought tolerance, index insurance, India, risk, uncertainty

## Abstract

In rainfed production systems throughout India, agricultural activities are dependent upon the summer monsoon, and any aberration in monsoon rainfall patterns can have severe consequences for rice production. There is considerable policy interest in designing programs to lower small-scale farmers’ exposure to these types of risk given the regularity with which adverse monsoon events occur. This paper introduces a field experiment conducted with two risk management options in the state of Odisha: a drought-tolerant rice cultivar; and a weather index insurance product designed to complement the performance of the cultivar. Uptake rates for the cultivar itself and for the joint product are compared across two years alongside an analysis of factors that predict uptake. Results indicate high levels of demand for both the products, albeit with a significant degree of price sensitivity. But this sensitivity is agnostic to the *nature* of price reductions, suggesting that public investments that lower the costs of risk management may be sufficient to encourage broad uptake, without necessarily relying upon distortionary subsidies as is so often done. Sustained demand between years one and two is primarily explained where individuals were indemnified in year one and had a large number of peers also purchasing the product.

## 1 Introduction

Reliance on the vagaries of the summer monsoon is an omnipresent risk for rainfed agricultural production in India. Delayed onset and early cessation of the monsoon, prolonged dry spells during the season, low overall volumes of rainfall, or excessive rainfall resulting in floods can all affect crop cultivation by reducing both total cultivated area and the resulting yields. At the farm and household levels, the risks associated with reliance on the monsoons can also drive smallholder farmers towards more conservative farming practices, under-investment in the use of modern inputs, sub-optimal livelihood strategies and long-term poverty traps (Hansen *et al.* 2004; Elbers *et al.* 2007; Hill 2009; Shiferaw *et al.* 2014; Emerick *et al.* 2016; Hill *et al.* 2019). At the national level, these risks can negatively affect rice production, prices and food consumption across the country.

Among the myriad production risks faced by farmers in rainfed geographies in India, droughts pose perhaps the greatest threat. Estimates suggest that more than 70 per cent of crop losses in India from 1985 to 2002 were attributable to droughts (Parchure 2002). Yet droughts are a very complex phenomenon, making them difficult to mitigate with improved cultivars, agronomic practices or financial instruments. The challenge to managing drought risk begins with the difficulty in defining drought. Contrary to popular belief, droughts are more than just periods of inadequate rainfall, although this is obviously a key contributing factor. Other factors include sustained high temperatures, high evapotranspiration rates via soils and plants, and other meteorological, hydrological and biological variables.

Given the magnitude of the impacts associated with drought risk, there is considerable interest among public policymakers in India and the broader development community in identifying strategies to help farmers effectively manage drought risk. India’s public agricultural research and extension system and its international counterparts have invested considerable resources during the past decade in breeding and distributing improved cultivars – particularly rice cultivars – that can withstand prolonged periods of drought (Pandey *et al.* 2012). They have also advanced crop and resource-management practices like zero tillage wheat cultivation and direct seeding of rice to reduce groundwater extraction, retain residual soil moisture and mitigate the impact of droughts (Erenstein *et al.* 2007).

There has been considerable interest in expanding the coverage of agricultural insurance within India. India has a long history with agricultural insurance, dating back to at least 1920. The largest program, a yield index crop insurance program known as the National Agricultural Insurance Scheme (NAIS), was introduced in 1999 and has grown to become the largest crop insurance program in the world, with more than 25 million participating farmers (World Bank 2012). Yet despite this size, this only represents less than approximately one fifth of the farmers in the country (Nair 2010a).^1^ Considerable issues remain, however, with the design of these insurance policies, their integration with credit provision, land titles and other program requirements, and the extensive subsidy regime required to make them marketable to smallholders. Furthermore, this program has been subject to considerable criticism for its reliance on crop cutting experiments as the basis for issuing payments, an exercise which significantly delays the claim settlement process.

Other programs, such as the Weather-based Crop Insurance Scheme (WBCIS), have been introduced. Since WBCIS was based on weather measurements instead of yield estimates from crop cutting experiments, insured farmers would have access to more timely indemnity payments. But despite the existence of these various schemes, coverage has remained low, with only about 23 per cent of cropped area covered by insurance. Increasing insurance coverage has thus become an important policy objective for Prime Minister Narendra Modi. In 2016, he introduced his own flagship program, Pradhan Mantri Fasal Bima Yojana (PMFBY), to supplant these other insurance programs. With this program, the Government of India aims to increase the insurance coverage to as much as half of India’s gross cropped area. Under PMFBY, farmers would pay incredibly low prices for insurance coverage: for *kharif* crops like rice, farmers only pay two per cent of the sum insured; the remainder will be subsidised by the government (shared between the central and respective state governments), with no cap on the amount of the subsidy. While it is not uncommon for governments to intervene in insurance markets and provide subsidies to increase coverage, the structure of PMFBY – with a variable and unlimited subsidy amount – seems particularly unsustainable. Furthermore, as has been advocated throughout the history of public economics, subsidies such as these entail considerable opportunity costs. Arguably, there are other public investments that could be made to support agriculture and rural development without distorting insurance markets.

All this suggests the need for innovation to increase the coverage of risk management products and services for millions of smallholder farmers across India, without simply relying on large government subsidies to prop up insurance markets (Nair 2010a). Consider, in particular, two products that have received considerable attention in both research and practice: drought-tolerant (DT) rice cultivars (Verulkar *et al.* 2010; Ward *et al.* 2014) and weather-based index insurance (WII) (Carter *et al.* 2014; Karlan *et al.* 2014; Hill *et al.* 2019). While both DT and WII enable farmers to eliminate some of their risk exposure, neither product provides a perfect solution to agricultural risk management. In an important theoretical contribution, Lybbert and Carter (2014) demonstrate that these two risk management instruments can pair well together and demonstrate that a bundled product consisting of *both* DT and WII can provide more comprehensive risk management product than either DT or WII on its own. In particular, they demonstrate how one could design a WII product to complement the performance of the DT cultivar under different drought conditions to provide farmers with an accessible and monotonically non-decreasing benefit stream irrespective of the degree of drought stress.

This is all particularly relevant in the eastern state of Odisha, one of India’s poorest and most vulnerable states. Odisha is often described as India’s ‘disaster capital’ owing to the frequency with which it has fallen victim to natural hazards such as cyclones, floods and droughts. The latter is particularly relevant for the present study. United Nations Development Programme (UNDP) estimates that Odisha experiences rainfall deficiencies of 25 per cent or more roughly once every 5 years (UNDP 2003). Pandey *et al.* (2007) estimates that the negative consequences of drought in Odisha include a 33 per cent reduction in gross cropped area and cropping intensity, a 41 per cent reduction in area, and a 60 per cent reduction in rice production during drought years relative to normal years, and total income losses of 26 per cent.

This paper provides some of the first empirical evidence on farmers’ demand for two drought risk management products: a recently released DT rice cultivar and the more comprehensive DT-WII risk management bundle. The paper makes several noteworthy contributions. First, we demonstrate how a bundled DT-WII product could be designed, highlighting the reduction in the cost of drought insurance achieved through the bundling approach. Second, we provide estimates for the price sensitivity of demand and other predictors of uptake for the DT cultivar and DT-WII product by drawing on survey data from a 2-year field experiment in which the DT cultivar and DT-WII product were marketed in randomly assigned villages across three drought-prone districts in Odisha. Third, and most importantly, we demonstrate that, although farmers may be price-sensitive, they are agnostic to the specific *nature* of price changes; specifically, framing a price reduction as a subsidy does not affect farmer demand. This has important implications for agricultural insurance policies in India, which are currently supported by large government subsidies. Our results suggest that these public funds might be more effectively allocated towards investments that can lower the cost of risk management in a more sustainable fashion and without distorting markets.

The remainder of the paper is organised as follows. Section 2 describes the drought risk management products examined in the present study and reviews the literature relevant to their development and evaluation. Section 3 details the study site, experimental design and sampling frame. Section 4 describes the data. Section 5 explains the empirical methods used to arrive at the estimates of determinants of product demand. Section 6 offers concluding remarks and ways forward for public policy.

## 2 Drought risk management products

### 2.1 Stress-tolerant cultivars

Until recently, drought tolerance has received relatively little attention from rice breeders, particularly with respect to rainfed lowland and upland production systems (Verulkar *et al.* 2010). Early breeding efforts designed to improve rice yields under drought conditions focused on the selection of secondary physiological or morphological traits, but were generally unsuccessful.^2^ Similarly, early molecular analyses of quantitative trait loci emphasised secondary traits that were not directly linked to improving yields under drought conditions (Price and Cutrois 1999). The crux of the challenge has been that efforts to enhance a particular physiological process in the plant to address drought have not necessarily resulted in higher yields under drought conditions (Fukai *et al.* 1999; Kumar *et al.* 2008). In addition, identifying important drought-tolerance traits is complicated by the fact that genotype–environment interactions are dependent on the timing and duration of drought stress and upon other co-occurring abiotic stresses such as heat stress (Price and Cutrois 1999).^3^

Despite these challenges, research on drought-tolerance traits and cultivars continues at both the global and national levels. Of particular note is the Stress Tolerant Rice for Africa and South Asia (STRASA) initiative, led by the International Rice Research Institute (IRRI) and AfricaRice, and supported by the Bill and Melinda Gates Foundation. Since its inception in 2007, STRASA has been actively developing and delivering stress-tolerant rice varieties across various risk-prone geographies. Operating through a participatory varietal selection model, STRASA has released several DT rice varieties in coordination with national agricultural research systems across South Asia. In India, the first of these DT varieties was *Sahbhagi dhan* (designation IR74371-54-1-1), released in 2010 by the Central Rice Research Institute. Results from multi-locational managed-drought screening trials spanning different drought severity levels (Table 1) show that when compared to other commonly cultivated varieties, *Sahbhagi dhan* suffered relatively small yield reductions under moderate and severe drought stresses, while also providing high yields under optimal (irrigated) conditions (Verulkar *et al.* 2010).

**Table 1:** Performance of *Sahbhagi dhan* relative to other rice varieties commonly grown in Odisha

Variety name	Days to 50% flowering	Grain yield (t ha^−1^)		
		Control (irrigated)	Moderate drought stress	Severe drought stress
*Sahbhagi dhan*	86	5.20	3.40	1.50
Swarna	107	5.30	2.20	0.60
Samba Mahusuri	107	4.80	3.20	0.10
MTU 1010	85	5.10	2.80	1.40
Vandana	66	2.60	1.70	0.60

However, these results should not imply that the adoption of a new DT rice cultivar will be either rapid or widespread across its target populations and geographies. Lybbert and Bell (2010) point out that the stochastic nature of droughts makes it difficult for farmers to learn about and assess the merits of a DT trait relative to a non-DT variety, or to assess the merit of a DT trait compared to traits that address more ubiquitous and frequent stresses, such as pests. Lybbert and Bell (2010) use the example of the insect-resistance trait conferred by the introgression of genes from *Bacillus thuringiensis* (Bt) into crops such as maize. In the absence of the Bt gene, infestations of the targeted pests are commonplace to the farmer, such that the introduction of a Bt cultivar provides observable benefits with immediate opportunities for farmers to learn about the trait’s effectiveness. Droughts, on the other hand, are more unpredictable and sporadic, such that the relative benefits of the DT trait may not be realised in any given year. This learning failure may be exacerbated by the fact that beyond some threshold drought stress level, farmers may not observe any benefits attributable to the DT trait (i.e. total crop failure) – a condition that is far less common for other traits such as biotic stress tolerance.

### 2.2 Weather index insurance

Agricultural insurance has existed in various forms since at least the early to mid-19^th^ century.^4^ Historically, most agricultural insurance programs have taken the form of multiple peril, indemnity-based crop insurance, which protect against crop damage arising from a number of different hazards (e.g. droughts, floods or hail) by providing payments on the basis of assessed on-farm losses (e.g. yields below a given percentage of historical average yield) (World Bank 2^011^). These traditional indemnity-based insurance programs are subject to a myriad of well-documented challenges, including information asymmetries in the form of moral hazard and adverse selection (Hazell 1992; Morduch 2006; Barnett *et al.* 2008; Miranda and Farrin 2012).^5^ Traditional indemnity-based crop insurance programs are also prone to other challenges, including high administrative costs (in particular, the cost of assessing losses), and the covariance of insured farmers’ risks that increases the insurers’ risk of insolvency or, at the least, increases their costs of reinsurance. All of these challenges are perhaps most pronounced in developing countries, where information asymmetries, knowledge gaps and other structural and operational issues are even more widespread. Moreover, despite a great deal of research, there remains relatively scant evidence to suggest that traditional crop insurance positively affects farmer welfare in either developed or developing countries (Hazell 1992; Skees *et al.* 1999; Smith and Watts 2009). Most crop insurance programs in the developed world have been supported by large government subsidies, and many developing countries exploring such programs are following suit.

In recent years, index insurance has emerged as an alternative to indemnity-based crop insurance because of its ability to reduce the information asymmetries and administrative costs associated with traditional crop insurance. Instead of basing indemnity payments on assessed losses, index insurance payouts are made based on the performance of an objective and easily verifiable index relative to some threshold. Typically, these indexes have been constructed to reflect weather conditions (in the case of WII) or the average yield in a particular geographic region. Since measurement and verification of losses at a farm or individual level are not required to determine payouts (and are thus independent of either farmer characteristics or farmer actions), index insurance significantly reduces the problems of moral hazard and adverse selection, as well as the delays and high costs of verification that are inherent in traditional crop insurance products (Barnett and Mahul 2007; Carter *et al.* 2014). Additional benefits of index insurance include its (relative) simplicity, the lack of context knowledge that is required for insurers and re-insurers in understanding their risk exposure, and low operational costs (Barnett and Mahul 2007).

The most prominent drawback of index insurance is the possibility that insured farmers experience losses but may not receive an insurance payout because of the imperfect correlation between weather conditions (e.g., rainfall) recorded at the weather station and production losses on the farm. The possibility that the insurance policy will fail to pay out when losses are experienced on the farm is referred to as ‘basis risk’.^6^ When a farmer purchases index insurance, she incurs the risk of paying a premium for an insurance policy that may not pay out even in the event of crop loss or yield reduction on their field. By the same token, the farmer also stands to benefit when adverse weather conditions are recorded at the weather station but not realised on their farm. Basis risk stems from two primary sources. First, it arises because of spatial heterogeneity in environmental conditions and the fact that weather variables may not be particularly strong proxies for on-farm yields or profits that such insurance is meant to protect (Rosenzweig and Binswanger 1993; Binswanger-Mkhize 2012). Second, to the extent that erratic rainfall patterns, especially droughts, are an important cause of on-farm losses, they are far from being the sole cause. Pests, diseases and animal attacks are among a host of other possible causes of crop loss.

Multiple studies have explored the uptake of weather index insurance (WII). For the most part, uptake has been low in the context of developing-country agriculture (Giné *et al.* 2008; Cai *et al.* 2009; Cole *et al.* 2013). Despite this, several recent studies have examined the determinants of insurance uptake and have identified constraints on both the demand and supply sides of the market. Not surprising, price frequently emerges as a key constraint in empirical studies of index insurance programs, though, as in the developed world, price subsidies seem to successfully stimulate demand (Mobarak and Rosenzweig 2012; Cole *et al.* 2013; McIntosh *et al.* 2013; Karlan *et al.* 2014; Hill *et al.* 2019). Other constraints include wealth and credit constraints (Giné *et al.* 2008; Clarke and Kalani 2011; Hill *et al.* 2013), education and understanding or familiarity with the insurance product (Giné *et al.* 2008; Gaurav *et al.* 2011; Cole *et al.* 2013; Hill *et al.* 2013), trust in the insurance provider (Giné *et al.* 2008; Cai *et al.* 2009; Dercon *et al.* 2011; Cole *et al.* 2013), and one’s own or one’s peers’ past experience with the insurance product (Cole *et al.* 2014; Karlan *et al.* 2014; Cai *et al.* 2015).

Platteau *et al.* (2017) extend these insights with a review on the demand for microinsurance which, while neither specific to agriculture nor index-based insurance, identifies several generalisable constraints that are worth noting. Platteau *et al.* (2017) point out that low uptake can typically be explained by characteristics of the potential insured (including such factors as risk and loss aversion, ambiguity aversion, present bias, low education levels and poor understanding of the insurance product), as well as serious challenges associated with providing high-quality insurance in many developing countries, including supply-side factors such as high insurance prices (related to, among other things, the costs of administration, risk premia and the costs of reinsurance), high transaction costs (e.g. the complexity of filing a claim), basis risk and poor quality of service.

### 2.3 Bundled DT-WII risk management product

Taken separately, abiotic stress-tolerant cultivars and WII can help small-holders mitigate risk, smooth consumption and invest in higher-risk, higher-return livelihood strategies. Empirical evidence from studies by Karlan *et al.* (2014), Emerick *et al.* (2016), and Hill *et al.* (2019) demonstrates that such effects are both observable at the farm and household levels, and appreciable in magnitude. Yet neither an improved cultivar nor an index insurance product offer a complete risk management solution, particularly when the hazard in question is drought in a smallholder-based rainfed production system. For example, *Sahbhagi dhan* may confer relative yield benefits vis-á-vis non-DT varieties under certain drought stresses, but there is some drought stress level beyond which the relative benefits are completely exhausted. A WII product, on the other hand, may provide monotonically non-decreasing benefit streams regardless of the degree of drought stress, but most products suffer from a nontrivial degree of basis risk, and most index insurance pilot projects to date have suffered from insufficient demand levels.

But by bundling these two products together – one technological, one financial – there is a possibility of overcoming their respective weaknesses through complementarity in design, as originally conceptualised in Lybbert and Carter (2014). Bundling an index insurance product with a DT cultivar ensures that the benefit stream is a monotonically non-decreasing function of drought stress, while bundling the DT cultivar with the index insurance product implies that a lower quantity of crop losses would need to be insured, thereby reducing the cost of the insurance. Given that plant breeding often requires 10–15 years of investment in research and development (McMullen 1987), it is a simpler proposal to design an insurance product that is optimised to complement the performance profile of the DT variety, rather than vice versa. Further, since most smallholders in developing countries are likely to be more familiar with improved cultivars than complex financial instruments, it makes sense from that perspective as well.

The bundled product introduced in the present study was specially developed by the research team to mimic what might someday be commercially available in the study area. We specified an insurance product that begins providing benefits at the approximate drought stress level at which the relative benefits of the DT cultivar vis-á-vis non-DT cultivars start to decline. This approach has some inherent appeal since the relative benefit profile of the bundled DT-WII product would be monotonically non-decreasing. Indeed, this is the approach that Ward *et al.* (forthcoming) use in specifying their hypothetical DT-WII product for their ex ante valuation in Bangladesh. Of course, this approach ignores the reality that, even though the *relative* benefits may be increasing in the presence of moderate droughts, the variety still suffers *absolute* yield losses under these drought conditions. In the present context, we modify this approach slightly by specifying our product such that the insurance product begins to provide benefits under moderate droughts, with an increased payout under severe drought stress. This design may be sub-optimal vis-á-vis the ideal design strategy laid out by Lybbert and Carter (2014), but because *Sahbhagi dhan* is a relatively new variety and there are insufficient data on the variety’s performance under a broad spectrum of environmental (including weather) and management conditions, insurance optimisation is not as straightforward in practice as theory might suggest. Details of the insurance product design can be found in Appendix S1A.

## 3 Research design

**Figure 1:**
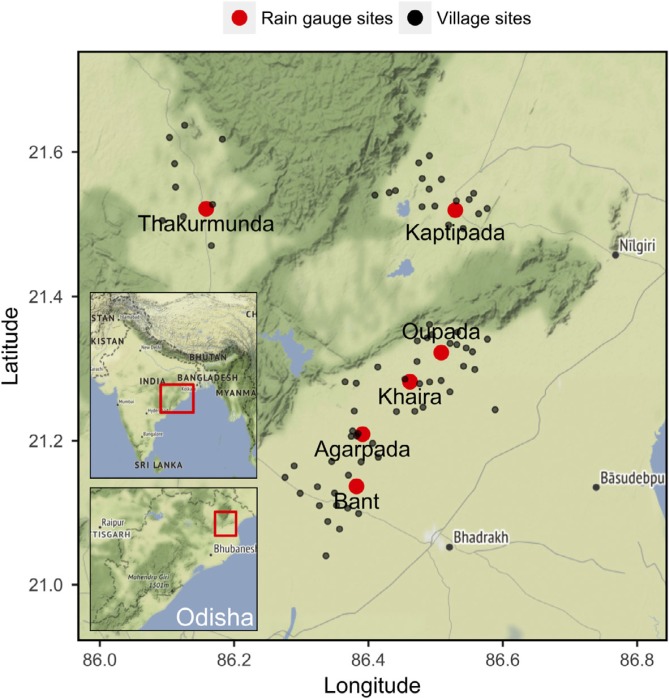
Location of sample villages and rain gauge installation sites. Source: Authors, using source map data provided by GGMAP (Kahle and Wickham 2013).

To better understand demand for our risk management products, we designed a multi-year randomised controlled trial to be conducted during the *kharif* (monsoon) rice-growing seasons in 2015 and 2016. The study area spans six blocks (sub-district administrative units) in three adjacent districts of Odisha: Khaira and Oupada in Balasore district, Agarpada and Bant in Bhadrak, and Thakurmunda and Kaptipada in Mayurbhanj (Figure 1).^7^ This region was identified for the study because farmers in this region predominantly grow rice and are extremely prone to drought. Importantly, *Sahbhagi dhan* was not available for purchase in local input markets in 2015 when this study was initiated, so the present study represents farmers’ first exposure to this drought-tolerant variety.

### 3.1 Sampling frame

Using population data for the six blocks in the study area from the 2011 Census, 111 villages were selected with probability proportional to size sampling.^8^ Each of these 111 villages was then randomly assigned to one of three groups: a DT treatment arm, a DT-WII treatment arm and a comparison group. Since the present study only focuses on product uptake among the two treatment groups (one offered only the DT variety and the other offered the DT-WII bundle), we limit our discussion to these groups. A total of 30 households were randomly sampled from each village, 20 of which were ultimately included in the sample, and 10 of which were retained as potential replacement households should any of the other 20 households be unavailable to participate or otherwise not qualify for inclusion.^9^ The resulting sample consisted of 1,460 households at baseline, with nearly all (1,416) of these households surveyed during follow-up in 2016 (97 per cent retention rate). Due to missing observations on some key explanatory variables, the sample ultimately used in the ensuing analysis consists of 1,347 observations in 2015 and 1,265 observations in 2016.

As shown in Table 2, 36 villages were offered DT rice seed and 37 were offered the DT-WII bundle (more details on the marketing of these products in Section 3.2 below).^10^ Households in the DT treatment group were permitted to purchase up to three units of seed, where each unit consisted of a 2 kg bag of *Sahbhagi dhan* seed. Households in the DT-WII treatment group were similarly permitted to purchase up to three units of the bundle, with each bundle consisting of one 2 kg bag of DT seed and one insurance policy meant to cover 0.1 acres of area under rice cultivation.^11^

**Table 2:** Breakdown of sample villages into treatment and control arms

	Treatment group	
	DT seed	DT-WII bundle
Number of villages	36	37
Unit of product offered	2 kg bag of seed	2 kg bag of seed & insurance policy for 0.1 acre of land
Maximum units offered	3	3

### 3.2 Marketing of the risk management products

In May 2015, following baseline data collection and prior to sowing for the 2015 *kharif* season, marketing agents employed by Balasore Social Service Society, a local nongovernmental organisation, were trained on the features of the two risk management products. In pairs, agents conducted marketing visits in each treatment village where they explained the product relevant for that specific village, drew a random discount in a public lottery, and facilitated the sale of DT seed DT-WII bundles. Three marketing sessions were conducted by agents with the 20 sample farmers in each village. The price of one 2 kg bag of seed was fixed at INR 80 (INR 40 per kg), whereas the price of one unit of the insurance policy varied by district due to different drought probabilities and different expected yield losses under drought conditions. After adding a 30 per cent premium to the actuarially fair cost of insurance, the cost of the complementary insurance component was INR 130 in Balasore, INR 150 in Bhadrak, and INR 100 in Mayurbhanj.^12^ To maximise the likelihood that price was not an inhibiting factor in the take up of treatment and to enable estimation of demand elasticities, random discounts of 10, 15, 20, 25 and 30 per cent were offered to participants based on village-level randomisation. In each treatment village, the discount was chosen by means of a public lottery conducted by the marketing agents at the culmination of the first marketing session. The randomly selected amount was then fixed for all future purchases in the given village. Although the discount rate was to be randomly selected, the *ex post* distribution of the discount rate was skewed in favour of higher discount rates.

In May 2016, following the second round of data collection and prior to nursery preparation for the *kharif* 2016 season, agents were once again trained and sent out to conduct three marketing sessions in each treatment village. In this second year, the actuarially fair price for the complementary insurance component was recalculated to factor in changes in the minimum support price and secular yield growth, both of which would contribute to interannual differences in the value of lost output under different drought scenarios. Each treatment group was randomly allocated into sub-groups in the second year, with households in one sub-group offered a discount (a fixed 20 per cent discount in the case of DT and a fixed 25 per cent discount in the case of DT-WII), and households in the other sub-group not offered a discount. For the former, the initial (pre-discount) prices were specially calibrated so that the effective price (after applying the discount) would be exactly the same as the price offered to the latter group (who were not offered a discount). In other words, this necessarily implies that the initial prices for the first group were artificially inflated (above and beyond the 30 per cent premium discussed above). The rationale for this ‘illusory’ discount is rather straightforward. There is considerable evidence that suggests that demand for insurance (including index insurance) is quite sensitive to price. As a result, governments in many developed countries have historically relied upon large subsidies to increase participation in crop insurance programs, and developing-country governments are following suit. There may be good reason for this: recent research into the behavioural dimensions of farmer decision-making has demonstrated that subsidies can provide an important inducement, particularly into making investments in agricultural technologies (Bell *et al.* 2017). Yet at the same time, large subsidies distort resource allocations and are almost always fiscally unsustainable. There is, therefore, a lingering question about the appropriate role for government interventions in encouraging uptake of risk management products like the ones explored here. Particularly relevant for the two products we consider in the present study, there are other potential roles for government interventions that can increase uptake in a more sustainable and less distortionary way. For example, governments could invest in seed research and development to generate drought-tolerant cultivars with even greater tolerance or resistance traits. This would have the dual benefit of increasing yield resilience while also lowering the cost of the complementary insurance component of a DTWII bundle like the one studied here.^13^ Alternatively, governments could invest in constructing weather stations or in developing remote sensing technologies that could result in lower basis risk, which could make WII products more attractive without necessarily increasing moral hazard. The introduction of this illusory discount – in conjunction with findings on the extent of price sensitivity from the first year of the pilot – will enable us to determine the extent to which uptake is driven by the bottom line alone versus the psychological inducement of a subsidy.

It has also often been observed that the demand for a particular risk management instrument can be influenced by sociological factors. For example, particularly in the case of insurance, which entails an up-front payment with the potential of a future return, participants may only engage in the market if they trust the insurer (Cole *et al.* 2013; Karlan *et al.* 2014; Hill *et al.* 2019). This may be particularly important in the context of rural India. In focus group discussions prior to the implementation of the present study, several farmers reported a series of NGOs that would visit their village, promise various services (including insurance), and then disappear with villagers’ money, never to be heard from again. Another sociological factor that may arise as a result of the manner in which the products were marketed (i.e. in group settings rather than on an individual basis) is social desirability bias. In other words, it is possible that observed participation in the risk management markets could reflect an individuals’ desire to be seen participating in the NGO’s activities, rather than truly reflecting utility-maximising behaviour.

The index insurance component was designed to provide coverage for 0.1 acres of area under rice cultivation, while the 2 kg bag of *Sahbhagi dhan* seed was recommended for cultivation on precisely the same area of land.^14^ The cost and structure of these insurance products across the three districts are shown in Table 3. The effective price of the 2 kg bag of seed was fixed at INR 80 in both years, reflecting the generally invariant price of rice seed – irrespective of variety – produced and disseminated by the public research system and state seed production facilities in Odisha. The actual price of the bundled product includes both the insurance component described above as well as the price of the seed, less any discounts as described above. Households were permitted to purchase up to three units (2 kg bags or bundles) – limited to the quantity needed to cover their landholding – so if a household maxed out their purchase quota they would be covering on average approximately 20 per cent of area under rice cultivation.

**Table 3:** Product price structure and discounts offered

Year 1	DT group		DT-WII group	
	(INR per 2 kg bag)	Discount (%)	(INR per bundle)	Discount (%)
Balasore	80	Random	210	Random
Bhadrak	80	Random	230	Random
Mayurbhanj	80	Random	180	Random

**Table:** 

Year 2	DT group 1		DT-WII group 1	
Balasore	100	20	270	25
Bhadrak	100	20	290	25
Mayurbhanj	100	20	230	25

**Table:** 

	DT group 2		DT-WII group 2	
Balasore	80	None	200	None
None Bhadrak	80	None	220	None
None Mayurbhanj	80	None	170	None

Note: DT, drought-tolerant; WII, weather-based index insurance. Source: Authors.

## 4 Data

### 4.1 Household data

Baseline and follow-up data were collected during household surveys conducted in March–April 2015 and February–March 2016, respectively. Table 4 presents a summary of household characteristics at baseline, including a comparison of means between the DT and DT-WII group. In the villages allocated to the DT-WII group, the concepts of insurance and basis risk were explained to farmers through an experiential learning exercise that was a variation of the one conducted by Marenya *et al.* (2014) in Malawi (see Appendix S1B). The concept of drought risk was explained to both the DT and DT-WII groups using a specially designed experiential learning exercise (see Appendix S1C). Each of these experiential learning exercises was carried out both at baseline and follow-up. In both years, therefore, these important concepts were explained to farmers prior to their purchase decisions so that participants would be well-informed.

**Table 4:** Characteristics of households in randomly allocated DT and DT-WII treatment villages

	(1) Full sample	(2) DT treatment arm	(3) DT-WII treatment arm	(4) DT-WII – DT difference
Age of household head	52.56 (0.37)	52.41 (0.51)	52.71 (0.54)	0.31 (0.75)
Gender of household head (male = 1)	0.95 (0.01)	0.96 (0.01)	0.94 (0.01)	−0.02 (0.01)
Household size	5.61 (0.07)	5.67 (0.10)	5.55 (0.09)	−0.12 (0.13)
Land owned (acres)	1.39 (0.04)	1.51 (0.05)	1.27 (0.05)	−0.24 (0.07)
Total paddy output (tonnes)	1.63 (0.04)	1.66 (0.06)	1.60 (0.05)	−0.05 (0.08)
Paddy yield (tonnes per hectare)	1.18 (0.01)	1.15 (0.02)	1.21 (0.02)	0.06 (0.03)
Trust Index	−0.03 (0.03)	0.03 (0.04)	−0.09 (0.04)	−0.12 (0.06)
Household savings (,000s)	2.46 (0.13)	2.38 (0.18)	2.54 (0.19)	0.16 (0.26)
Asset index	−0.08 (0.03)	−0.17 (0.04)	0.01 (0.04)	0.17 (0.06)
Agricultural input expenditures (2014; ,000s)	18.35 (0.37)	18.32 (0.54)	18.38 (0.50)	0.06 (0.74)
Household annual consumption expenditures (,000s)	135.24 (2.31)	128.73 (3.25)	141.47 (3.27)	12.74 (4.61)
Time discount rate	3.58 (0.07)	3.94 (0.12)	3.24 (0.09)	−0.70 (0.15)
Ambiguity averse (= 1)	1.51 (0.01)	1.52 (0.02)	1.49 (0.02)	−0.02 (0.03)
Risk aversion coefficient	1.73 (0.03)	1.86 (0.05)	1.60 (0.05)	−0.26 (0.07)
Subjective beliefs about length of dry spell (mean)	22.36 (0.23)	22.00 (0.34)	22.71 (0.31)	0.72 (0.46)
Subjective beliefs about length of dry spell (SD)	75.49 (0.90)	73.91 (1.29)	77.00 (1.27)	3.08 (1.81)
Distance to rain gauge (km)	5.95 (0.90)	6.71 (0.11)	5.23 (0.14)	−1.48 (0.18)
No. observations	1,348	659	689	

Note: Monetary figures in Indian rupees (INR). Differences in column (4) are based on slope coefficient estimates from linear regressions of the form *x*_ij_ = α + β*T* + ε_*ij*_, where *x*_ij_ is the characteristic over which balance is being tested (i.e. the variable described in the row header) and *T*_i_ is an indicator variable capturing the difference in random assignment between DT and DT-WII groups. Standard errors in parentheses. DT, drought-tolerant; WII, weather-based index insurance.

Source: The authors.

Additionally, a simple laboratory-in-field experiment was used to elicit farmers’ subjective beliefs about the length of the longest dry spell during the subsequent *kharif* season. These experiments were similar in scope to those introduced in Luseno *et al.* (2003) and Lybbert *et al.* (2007). Farmers were given 20 beans and a sheet of paper with six bins, each representing a range of days, specifically, 0–7, 8–14, 15–21, 22–28, 29–35 and 36 or more days. Farmers were then asked to allocate the 20 beans across these bins based on how likely they perceived the longest dry spell to be in the coming *kharif* season. Prior to playing this exercise, farmers undertook a series of similar – but simple – bean allocation exercises to ensure that they understood the concept of probability. Following data collection, the research team was able to take the recorded allocation of the beans across the six bins and calculate the moments of each individual’s drought risk belief distributions by treating each bin as a uniform distribution defined over its range, with each such uniform distribution in turn being a component of a stepwise distribution. The probability that the farmer places on the bin functions as a weight on the corresponding distribution, and since each component is a uniform distribution, we rely on the assumption that farmers place equal probability on each value of the corresponding bin.^15^ We calculate both the mean and the standard deviation of individuals’ belief distributions, with the mean serving as a clear proxy for individuals’ subjective expectation about the severity of the longest dry spell in the forthcoming season, and the standard deviation providing a proxy for the uncertainty around this expectation. While it is possible that individuals’ subjective expectations could evolve over time (e.g. as one gathers more observations with which to update beliefs), we treat these belief parameters as fixed, since they were only collected during endline data collection.

### 4.2 Rainfall data

Because one measure of the quality of a WII product is the degree to which it minimises basis risk – an important source of which is the mismatch in weather conditions on a farmer’s field and those at the location where index measurements are collected – we measured rainfall using rain gauges installed in the vicinity of block headquarters in each of the six blocks (refer again to Figure 1).^16^

Actual rainfall data was collected during the summer monsoon in 2015 and 2016, specifically from June 15 to October 15 at all six rainfall measurement stations. In *kharif* 2015, moderate droughts were recorded in Bant and Khaira, and a severe drought was recorded in Thakurmunda (dry spell of 25 days). In *kharif* 2016, only a single moderate drought was observed in Thakurmunda.

While there is some variation in households’ distances from the rain gauges relevant to their insurance product, the vast majority of households were within 10 km from the rain gauge site, with no household more than approximately 15 km from the gauge. While farmers might not have known exactly where the gauges were installed, they were informed that the gauges would be installed in their block headquarters, so their distance from the block headquarters (proxied in this case by the more objective measurement of the distance from their homestead to the actual gauge site) may prove an important determinant of their risk management product purchase decisions.

## 5 Demand for drought-tolerant rice and WII

### 5.1 Empirical approach

When modelling the demand for these two risk management products, an important factor that merits careful consideration is the high percentage of non-purchases, revealed as zeros in the data. While zeros potentially result from several different reasons, their interpretation has a crucial bearing on how the demand relationship is subsequently estimated. Given the manner of control we had over data collection efforts, we posit that these zeros result from corner solutions in our participants’ utility maximisation problem. As such, we consider demand for our risk management products to be a sequential process in which there are ultimately two decisions that the participating individual makes. First, the individual considers whether to participate in the market for these risk management products, which is a binary decision. Second, those individuals who enter the market decide how many units to purchase, which is a count or semi-continuous decision. It is assumed that the decision on how much to purchase is made *after* the decision to participate in the market, rather than simultaneously. To better understand this sequential decision-making process, we estimate a two-part model with a linear probability model followed by least squares.^17^ We pool data from the DT and DT-WII groups and estimate each of these regression models separately for each year (2015 and 2016).

In the first year (2015), since farmers had no prior experience with the products (either their own experiences or the experiences of their peers) that could potentially condition the purchase decisions, the determinants of product uptake include the effective product price, relevant household- and farm-level characteristics and individual preferences that may influence risk management decisions. The binary market participation regression equation can be written as:

(1) $$y_{i1}=\alpha_{11}+\theta_{11}p_{i1}+\xi_{11}T_{i}+\lambda_{11}^{1}y_{-i,1}^{1}+\lambda_{11}^{2}y_{-i,1}^{2}+\sum_{k=1}^{K}\gamma_{k1}z_{ik}+v_{i1},$$

where *y*_*i*1_ is a binary indicator variable equal to 1 if individual *i* was observed to have participated in the risk management market (i.e. she purchased either DT or DT-WII) in 2015, *p*_*i*1_ is the effective price of the risk management product (gross or market price less any subsidy), *T* is an indicator variable equal to 1 if the household belonged to a village randomly assigned to the DT-WII treatment arm, $y_{-i,1}^{j},j\in \left\{1,2\right\}$ are measures of the proportion of farmer *i*’s social network (i.e. other members of his or her village, excluding farmer *i*) who participated in the DT (*j* = 1) or DT-WII (*j* = 2) market in 2015, and *z*_*i*1_ = 〈*z*_*i*1_, *z*_*i*2_, …, *z*_*iK*_〉 is a vector of individual-, household- and farm-level characteristics specific to decision-maker *i*.^18^ The randomisation of the discount rate in the first year implies that individuals faced a range of effective prices, which permits us to assess the price sensitivity of market participation. Then, the quantity purchased among those participating in the market is estimated as:

(2) $$q_{i1}=\alpha_{21}+\theta_{21}p_{i1}+\xi_{21}T_{i}+\lambda_{21}^{1}y_{-i,1}^{1}+\lambda_{21}^{2}y_{-i,1}^{2}+\overset{K}{\underset{k=1}{\sum }}\beta_{k1}z_{ik}+\epsilon_{i1},$$

where *q*_*i*1_ is the number of units (2 kg bags of DT seed or bundles of DT-WII consisting of a 2 kg bag of DT seed and a single WII policy), and $p_{i},T,y_{-i,1}^{j}$, and *z*_*i*1_ = 〈*z*_*i*1_, 1, *z*_*i*2_, 1, …, *z*_*iK*_, 1〉 are defined as before.^19^ In Equations (1) and (2), the m_1_ and e_1_ terms are idiosyncratic error terms that are independent from each other for each individual *i*, but given the clustered nature of the interventions, we adjust them for within-village correlations.

In the second year (2016), we allow for farmer decisions to be conditioned by past experiences. Additionally, although the effective price for all participants was the same in 2016 (within products), the introduction of the randomly allocated ‘illusory’ discount affords us the opportunity to understand the extent to which farmers’ purchase decisions are driven by preferences for risk management versus preferences for subsidies. To study the market participation decision in 2016, we consider the following equation:

(3) $$\begin{array}{ccc} y_{i2} & = & \alpha_{12}+\psi_{1}^{1}y_{i1}^{1}+\psi_{1}^{2}y_{i1}^{2}+\xi_{12}T_{i}+\eta_{1}d_{i1}+\mu_{1}^{1}\left(y_{i1}^{1}\times d_{i1}\right)+\mu_{1}^{2}\left(y_{i1}^{2}\times d_{i1}\right)\\ & & +\tau_{1}^{1}y_{-i,1}^{1}+\tau_{1}^{2}y_{-i,1}^{2}+\lambda_{12}^{1}y_{-i,1}^{1}\lambda_{12}^{2}y_{-i,2}^{2}+\zeta_{1}^{1}\left(y_{-i,1}^{1}\times d_{i1}\right)\\ & & +\zeta_{1}^{2}(y_{-i,1}^{2}\times d_{i1}\phi_{1}s_{i}+\overset{K}{\underset{k=1}{\sum }}\gamma_{k2}z_{ik}+v_{i2}, \end{array}$$

where $y_{i}^{j}$ and $y_{-i}^{j},j\in \left\{1,2\right\}$ are defined as before, but now indexed over periods 1 and 2 (2015 and 2016, respectively); *d*_*i*1_ is a dummy variables that equals 1 if the rainfall conditions in the block were consistent with drought conditions (as defined in the insurance contracts) in 2015, and zero otherwise; *s*_*i*_ is a dummy variable that equals 1 if the village was randomly allocated to receive an subsidy, and zero otherwise; and *z*_*i*2_ = 〈*z*_*i*1_, *z*_*i*2_, …, *z*_iK_〉 is a vector of individual-, household- and farm-level characteristics specific to decision-maker *i*. The interaction terms ($y_{i1}^{j}\times d_{i1}$) and ($y_{-i,1}^{j}\times d_{i1}$) allow us to capture the effects of one’s own and one’s village network’s experiences with the drought risk management products during drought scenarios (i.e. scenarios in which some benefits from the drought risk management products are presumably reaped), respectively. *A priori*, we would expect that having purchased a drought risk management product in 2015 (*y*_*i*1_ = 1) and having experienced a drought during that year (*d*_*i*1_ = 1) would have a positive effect on risk management purchasing decisions in 2016. For farmers who purchased a DT-WII product in 2015 but who resided in blocks in which the rainfall conditions were not consistent with drought conditions (and hence did not receive an insurance payout), we would expect these experiences to have a neutral to slightly negative effect on their drought risk management purchasing decisions in 2016.

(4) $$\begin{array}{ccc} q_{i2} & = & \alpha_{22}+\psi_{2}^{1}y_{i1}^{1}+\psi_{2}^{2}y_{i1}^{2}+\xi_{22}T_{i}+\eta_{2}d_{i1}+\mu_{2}^{1}\left(y_{i1}^{1}\times d_{i1}\right)+\mu_{2}^{2}\left(y_{i1}^{2}\times d_{i1}\right)\\ & & +\tau_{2}^{1}y_{-i,1}^{1}+\tau_{2}^{2}y_{-i,1}^{2}+\lambda_{22}^{1}y_{-i,2}^{1}+\lambda_{22}^{2}y_{-i,2}^{2}+\zeta_{2}^{1}\left(y_{-i,1}^{1}\times d_{i1}\right)\\ & & +\zeta_{2}^{2}\left(y_{-i,1}^{2}\times d_{i1}\right)+\phi_{2}s_{i}+\sum_{k=1}^{K}\gamma_{k2}z_{ik}+v_{i2}, \end{array}$$

where *q*_*i*2_ is measures of the number of units purchased by household *i* in 2016, and all other terms are defined as before.

Before proceeding with describing our econometric results, it is important to recognise that, due to correlations between unobserved individual characteristics that would similarly affect purchase decisions in both 2015 and 2016, the 1-year lagged purchase variables are likely not exogenous and thus should be instrumented. Karlan *et al.* (2014) examined learning and social influences in a multi-period study of insurance demand, instrumenting for the lagged binary purchase decision and the interaction between the lagged binary purchase decision and the occurrence of a drought with the randomly assigned price from 2015 and the interaction between the random price from 2015 and the occurrence of a drought. We attempted to follow a similar approach in addressing the endogeneity of past purchases, but due to the rather weak correlation between price and demand in 2015, we find this instrument to be quite weak. As is well-established in the statistics and econometrics literature (Bound *et al.* 1994), when there is a weak correlation between the instrument and the endogenous variable, even a weak correlation between the instrumental variable and the error term can seriously bias estimates and do more harm than good. Consequently, we have chosen to not present the results of a model using instrumental variables, but instead rely on least squares estimates, noting that (assuming a positive effect of lagged purchase decisions on contemporaneous decisions) the estimates of experiential effects will be biased upward.

### 5.2 Results

In the first year, we find relatively high uptake for both products: 63.1 per cent of treated farmers offered the DT cultivar purchased seed, while 58.7 per cent of treated farmers offered the DT-WII product purchased the product. In the second year, the uptake rates of both products fell to 45.3 per cent uptake of the DT cultivar and 35.7 per cent uptake of DT-WII product.

**Figure 2:**
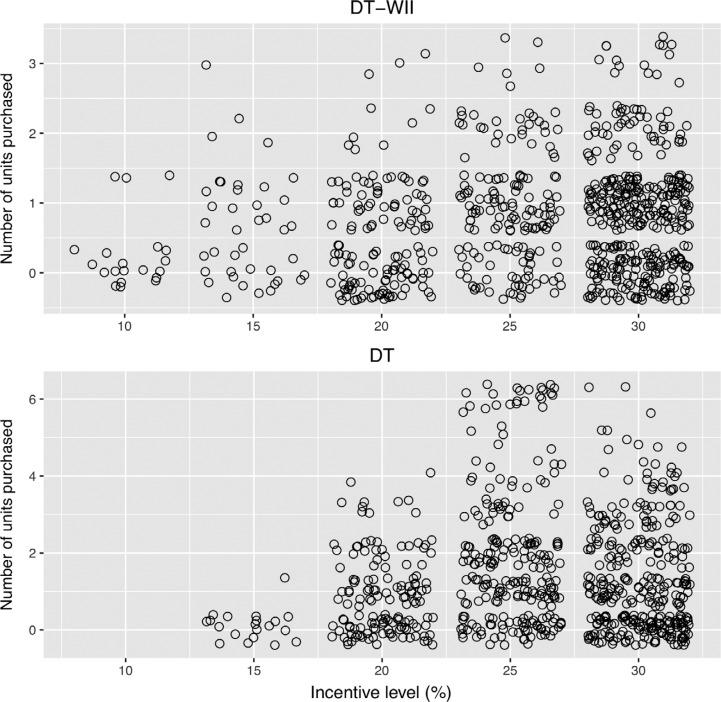
Scatter plot of purchased DT-WII and DT by incentive type in 2015. Note: Farmers did not purchase negative amounts of the product; the scatter plot is ‘jittered’ to avoid overplotting. DT, drought-tolerant; WII, weather-based index insurance. Source: Authors.

**Figure 3:**
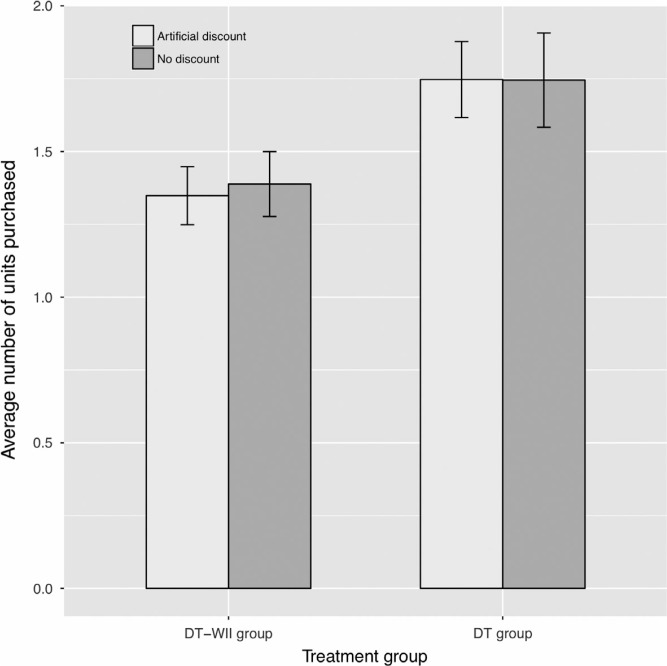
Bar plot of purchased DT-WII and DT by incentive type in 2016. Note: Vertical bars represent a 95 per cent confidence interval. DT, drought-tolerant; WII, weather-based index insurance. Source: Authors.

Figure 2 plots the number of units purchased (for both DT and DT-WII) against the five randomly offered discount levels in 2015, with DT uptake in the upper panel and DT-WII uptake in the lower panel. For both the DT cultivar and the DT-WII product, there is greater heterogeneity in purchasing behaviour at higher discounts (lower prices), though across both products the absolute degree of heterogeneity in purchasing behaviour is thin. Figure 3 presents the difference in average demand among those allocated to the artificial discount group and those offered the fixed price for both DT-WII and DT treatment arms. Uptake patterns in the second year indicate no significant differences between these two groups for either product. These results suggest that although demand for both risk management products appears to be somewhat price-sensitive, the manner in which the lower price is framed (e.g. if the effective price is the result of a subsidy) does not affect demand. While these visualisations are only bivariate representations and do not control for the influences of other factors, this is a first indication that demand might be commensurate with pricing that approaches actuarially fair levels.

Table 5 presents estimation results from the two-part model for DT and DT-WII uptake in the first year of the experiment. Column 1 presents linear probability model estimates of the determinants of market participation (a binary choice), while column 2 reports least squares estimates of the intensity of market participation (i.e. the number of units purchased). Few of the individual-, farm- or household-level characteristics that we might expect to determine DT or DT-WII demand are particularly useful in explaining either participation in the market or the intensity of participation. Although DTWII uptake rates are lower on average, we see that market participation is actually higher in villages that were offered the DT-WII product than in those only offered the DT variety, once we control for the influence of other factors. We do not see this effect carry over to the intensity of market participation, but this may be more a result of limited variation in the number of units purchased than anything. There are potentially a couple of reasons why there was limited variation in the intensity of market participation during this first year (discussed in greater detail below), but this result might suggest that farmers perceive the benefits from the bundled product and that this is an important determinant of demand for the bundled product, once we account for price and other factors.

**Table 5:** Two-part model regression results: market participation and uptake for DT and DTWII in 2015

	LPM: Participation	Least squares: Total units
Effective price of risk management product (after discount)	0.002 (2.552)	0.001 (0.250)
Allocated to DT-WII group	0.371 (3.095)	0.066 (0.074)
Share in village that purchased DT-WII (2015)	0.498 (4.910)	0.008 (0.032)
Share in village that purchased DT (2015)	0.802 (13.911)	1.324 (1.610)
Time discount rate	0.006 (1.195)	0.004 (0.304)
Is ambiguity averse	0.006 (0.271)	0.030 (0.365)
Risk aversion coefficient	0.007 (0.684)	0.043 (1.044)
Trust index	0.003 (0.231)	0.041 (1.212)
Distance from homestead to rain gauge	0.008 (1.863)	0.017 (0.226)
Distance to rain gauge × Allocated to DT-WII group	0.001 (0.243)	0.000 (0.002)
*R*^2^	0.11	0.26
*N*	1,347	827

Note: *t*-statistics based on standard errors clustered at the village level in parentheses. Regressions contain intercept terms and also control for household head age and sex, household size, land owned (acres), total input expenditures in 2014, total rice output (tonnes), savings (000s), assets (asset index) and annual consumption expenditure. DT, drought-tolerant; WII, weather-based index insurance.

Source: Authors.

Market participation is more sensitive to price than the intensity of participation. We estimate that a INR 10 increase in the effective price would reduce the probability of market participation by approximately 2 per cent. Conditional on participating in the market, however, price changes have relatively little effect on the number of units or bundles purchased. In addition to relatively inelastic demand among those interested in participating in the market, there are likely two other factors that contribute to this effect, both of which reflect a form of rationing. First, for various reasons – including budget limitations and a need for the program implementers to maintain solvency in the midst of considerable covariate risk – farmers were exogenously restricted in the number of units they were allowed to purchase. Second, and perhaps more importantly, the DT-WII represents a very new and arguably complex product – and, importantly, one that entails at least some basis risk – that farmers have no prior experience with, and therefore, farmers may have purposefully limited their exposure to the DT-WII product. Both of these factors might be expected to wane at scale and over time. At a larger scale and with adequate reinsurance, the provider rationing described above may not be a limiting factor. Similarly, as farmers gain more experience with and understand the nuances of this type of risk management product more, they may be less likely to ration their own exposure to the product and its complexities.

For both the DT cultivar and the DT-WII product, the share of other farmers in the village who purchased these products has a large and positive on the decision to purchase but, once again, not with the number of units purchased. We are cautious to ascribe causality here since we cannot disentangle whether household *i*’s behaviour directly influences (or is directly influenced) by the behaviour of the members of *i*’s village, or whether these behaviours are simply correlated because of other factors, either observable or unobservable (Manski 1993). Suffice it to say, however, the persistent positive effect suggests some level of peer influence even if this influence is limited in the first year to encouraging market participation, and not the intensity of market participation.

We find some evidence (albeit relatively weak) that a higher degree of impatience (indicated by a higher discount rate) is positively associated with the decision to purchase but not the decision of how many units to purchase. This implies that farmers who value present consumption more than future consumption are more likely to purchase these risk management products. At first glance, this may seem somewhat surprising, given that such purchases entail a current expenditure with an uncertain future return. Nevertheless, this phenomenon has been observed elsewhere in regard to insurance purchases (Ito and Kono 2010; Platteau *et al.* 2017; Hill *et al.* 2019).^20^ These findings may suggest that farmers are aware of their own lack of self-control and thus purchased the DT-WII risk management product to manage the self-control problems, although other, equally valid, explanations might be worth exploring.

For the second year of the experiment, Table 6 presents the results from the demand estimations for DT and DT-WII. As before, column 1 presents estimates from a linear probability model corresponding to the binary market participation, while column 2 reports estimates from a least squares regression on the intensity of market participation. Unlike what was found in year 1, there does not appear to be a different rate of uptake among households allocated to the DT-WII treatment after controlling for other factors. One of the principal results is the very small coefficient estimate associated with the artificial discount. This non-effect is ubiquitous in the 2016 estimates, regardless of whether we are talking about market participation or the subsequent determination of intensity of market participation. The evidence fairly strongly implies that farmers are not drawn to purchase these risk management products simply due to the presence of a subsidy or discount. Rather, although the results from 2015 suggest they are somewhat sensitive to price, there is also considerable evidence that farmers in our sample area are willing to pay more than the actuarially fair price and would not need to rely upon a subsidy to encourage their participation in risk management markets.

**Table 6:** Two-part model regression results: market participation and uptake of DT and DTWII in 2016

	LPM: Participation	Least squares: Total units
Purchased DT-WII (2015)	0.058 (1.287)	−0.173 (2.083)
Purchased DT (2015)	0.217 (3.079)	0.303 (1.629)
Allocated to DT-WII group	0.011 (0.185)	−0.195 (0.539)
Drought occurred (2015)	−0.093 (1.791)	−0.281 (0.804)
Purchased DT (2015) × Drought occurred (2015)	−0.082 (0.924)	−0.352 (1.484)
Purchased DT-WII (2015) × Drought occurred (2015)	0.234 (2.919)	0.402 (2.910)
Share in village that purchased DT (2015)	−0.139 (1.177)	−0.883 (1.476)
Share in village that purchased DT (2016)	0.694 (7.388)	0.439 (0.812)
Share in village that purchased DT-WII (2015)	−0.070 (0.836)	−0.554 (2.013)
Share in village that purchased DT-WII (2016)	0.801 (10.997)	0.165 (0.586)
Share in village purchased DT (2015) × Drought occurred (2015)	0.199 (1.772)	0.940 (1.663)
Share in village purchased DT-WII (2015) × Drought occurred (2015)	0.019 (0.145)	0.550 (1.053)
Allocated to artificial discount group	0.005 (0.308)	0.017 (0.156)
Distance from homestead to rain gauge	0.006 (1.446)	0.007 (0.272)
Distance to rain gauge × Allocated to DT-WII group	−0.003 (0.546)	0.005 (0.169)
*R*^2^	0.23	0.20
*N*	1,265	513

Note: *t*-statistics based on standard errors clustered at the village level in parentheses. Regressions contain intercept terms and also control for household head age and sex, household size, land owned (acres), total input expenditure in 2015, total rice output (tonnes), savings (000s), assets (asset index), annual consumption expenditure. DT, drought-tolerant; WII, weather-based index insurance.

Source: Authors.

Farmers’ activity in 2016 seems to be heavily influenced by experiences from 2015. Farmers who purchased DT during 2015 were more likely to purchase DT again in 2016, though farmers who purchased DT in 2015 and resided in blocks that were affected by droughts were actually about 8 per cent less likely to purchase DT again than farmers who resided in blocks not affected by droughts, though still over 13 per cent more likely than farmers who had not purchased DT in 2015. The lower uptake rate vis-á-vis DT-adopters who did not experience a drought in 2015 likely reflects the fact that the DT is not impervious to droughts, and absolute yields may still decline even when the yields relative to non-tolerant varieties may not decline as much. There is also some evidence of learning from the experiences of others in their communities. For example, although there is no evidence of peer experiences influencing DT market participation in blocks unaffected by droughts (and, indeed, the point estimate associated with this effect is negative), there is evidence of a positive influence of peer’s experiences in drought-affected blocks. For a given share of village peers who purchased DT in 2015, observing peer’s fields during drought conditions in 2015 increased DT market participation by about 30 per cent.

Similarly, experiences with DT-WII in 2015 generally have a significant effect on DT-WII uptake in the second year, though it depends critically on whether the household experienced a drought during 2015 (and thus received a payout from the insurance policy). If the household purchased DT-WII and did not experience a drought during 2015 (or, at least there was not a drought recorded at the block weather station site), then although there is no strong evidence of an effect on participation in the DT-WII market, they purchased 0.17 fewer units than farmers who had not purchased DT-WII during 2015, conditional upon participating in the DT-WII market. Farmers who purchased DT-WII during 2015 and lived in blocks that were affected by droughts in 2015 were 29 per cent more likely to purchase DT-WII again in 2016 and purchased more than 0.2 additional units of DT-WII in 2016. In some regards, this effect is discouraging, since it may point to receipt of an insurance payment as the primary motivator behind subsequent risk management decisions, particularly in light of the scant evidence of positive effects from having cultivated DT in the midst of a drought during 2015 on subsequent DT purchases.

Consistent with findings from 2015, we find evidence of a generally strong peer influence on participation in the DT and DT-WII products, with additional evidence of peer influences on the intensity of uptake of the DTWII product in 2016. Rather than this peer influence arising as a result of learning from one’s neighbours, the evidence seems to suggest that the primary driver behind this peer effect is due to a form of peer pressure or herd behaviour during the product marketing sessions. A plausible explanation behind this is that there is some social desirability about participating in the NGO’s activities: in other words, farmers in the sample villages want to be seen purchasing the DT and DT-WII products being marketed by the NGO, even though there is evidently not significant pressure on them to participate to the maximum extent possible (evidenced by the limited evidence of social influences on the intensity of participation).

Some of these findings – éspecially those related to previous experience with the drought risk management tools in 2015 – merit further explanation. In particular, what about farmers’ experiences may be driving the observed demand effects during 2016? In estimating the local average treatment effect (LATE; see Imbens and Angrist 1994) of the DT and DT-WII products on rice yields during the 2015 season (Table D1 in Appendix S1D), farmers who cultivated the DT variety and resided in blocks affected by droughts had higher yields than farmers in the control group (0.28 tonnes per acre more in DT villages, and 0.34 tonnes per acre more in DT-WII villages). Interestingly, households in DT-WII villages who cultivated the DT variety and yet were not affected by droughts saw a reduction in yields (0.39 tonnes per acre), which may explain the lower participation and intensity of DT-WII market activity among these farmers in the second season. We also note that – as expected – droughts had a strong negative impact on yields during the 2015 season (a 0.47 tonne per acre reduction vis-á-vis the average in blocks not affected by drought). The DT variety helped to offset some of these yield losses, providing relative yield gains (i.e. a reduction in yield losses) of approximately 0.07 tonnes per acre among households who cultivated the DT without complementary insurance, and absolute yield gains of approximately 0.73 tonnes per acre among households who cultivated DT and also had additional insurance.

Some of the inconsistencies (in particular those between farmers who purchased DT-WII in blocks not affected by droughts compared with those who purchased DT-WII in blocks that were affected by droughts) may arise due to some confusion about appropriate management of the DT variety. During the endline survey, we gathered information from respondents regarding their understanding of the characteristics of *Sahbhagi dhan*. In particular, we provided respondents with a series of statements about *Sahbhagi dhan* and asked them to respond to each statement. These results are reported in Table 7. While the vast majority of respondents were able to correctly identify that *Sahbhagi dhan* could withstand long dry spells and was a short-duration variety, relatively fewer were able to correctly identify *Sahbhagi dhan* as a high yielding variety even in the absence of drought conditions.

**Table 7:** Participants’ understanding of the features of *Sahbhagi dhan*

Statement	Proportion with correct response (%)		
	Full sample	DT group	DT-WII group
Sahbhagi dhan can withstand long dry spells	85.210 (0.010)	88.050 (0.012)	82.450 (0.014)
Sahbhagi dhan is a short-duration variety	94.460 (0.006)	94.400 (0.009)	94.530 (0.009)
If there is no drought, Sahbhagi dhan has the ability to produce high yields	57.610 (0.013)	61.800 (0.019)	53.530 (0.019)

Note: Standard errors of empirical proportions in parentheses. DT, drought-tolerant; WII, weather-based index insurance.

Source: Authors.

We also asked respondents to indicate how they managed their crops differently (if at all) if they cultivated the *Sahbhagi dhan*. One common criticism of insurance is that it induces moral hazard, and one of the touted advantages of WII is that it should – in theory – reduce the risk of moral hazard, since insurance payouts are not based on assessed crop losses, but are instead based on measured weather conditions geographically removed from the insured farmer. Quiggin (1992) demonstrated that crop insurance could result in both positive and negative negligence (Roumasset 2006). Negative negligence is the tendency for farmers to overuse risk-reducing inputs, while positive negligence is the tendency for farmers to overuse inputs that are yield-increasing in ‘good’ states of the world and yield-decreasing in ‘bad’ states of the world. As seen in Table 8, we see some evidence that the risk management products examined in the present study avoid both of these types of negligence. While approximately 20 per cent of farmers indicated that they did nothing different in managing their *Sahbhagi dhan* crop relative to their other varieties, a nontrivial portion of farmers also indicated several managerial decisions consistent with a reduced demand for risk-reducing inputs (e.g. herbicides, pesticides and irrigation). And further, there is little evidence that farmers increased their use of inputs with positive marginal products *only* in ‘good’ states of the world (e.g., fertiliser) but negative marginal products in less optimal conditions, which are a frequent occurrence in drought-prone areas like those in the present study. But while this may indicate avoided positive negligence, this also necessarily limits farmers’ exposure to these positive marginal products under good growing conditions.

**Table 8:** Crop management changes in cultivating *Sahbhagi dhan*

Crop management	Midline (%)			Endline (%)		
	Full sample	DT group	DT-WII group	Full sample	DT group	DT-WII group
More labour	0.95	-	1.84	1.84	2.40	1.14
Less labour	18.80	14.60	22.73	32.50	32.63	32.32
More fertiliser	5.44	6.85	4.13	1.01	0.90	1.14
Less fertiliser	46.57	44.20	48.77	52.76	50.60	55.51
More irrigation	2.61	2.96	2.29	0.50	0.60	0.38
Less irrigation	37.53	39.17	35.99	47.91	44.01	52.85
More herbicide	1.07	0.49	1.61	0.84	0.60	1.14
Less herbicide	14.78	14.63	14.91	20.10	20.96	19.01
More pesticide	1.43	0.49	2.30	2.85	1.80	4.18
Less pesticide	21.67	23.97	19.50	32.16	30.24	34.60
Sown earlier	5.91	5.38	6.41	2.18	2.40	1.90
Sown later	0.24	0.25	0.23	2.68	2.99	2.28
Transplanted earlier	12.13	10.84	13.33	10.22	9.88	10.65
Transplanted later	9.47	10.05	8.92	13.23	12.28	14.45
Harvested earlier	43.76	45.17	42.44	39.70	37.43	42.59
Harvested later	2.50	2.71	2.30	1.84	1.20	2.66
Did nothing different	19.18	17.44	20.77	20.94	22.75	18.63
Other	2.84	3.17	2.53	2.35	2.40	2.28

Note: Percentages may sum to > 100% because participants were allowed to select multiple alternatives. DT, drought-tolerant; WII, weather-based index insurance.

Source: Authors.

## 6 Concluding remarks

Indian agriculture remains highly susceptible to weather-related production risks, most notably droughts and floods. This is especially true in rainfed production systems, which accounts for approximately 60 per cent of the gross cropped area in India (Nair 2010b). When these risks are realised, there can be large impacts on crop production, but even when droughts and floods do not materialise the risk of their occurring entices farmers to opt for conservative farming practices and livelihood strategies. But despite the existence of various schemes over the years, coverage has remained dismally low. In 2016, the Government of India launched a new flagship program, Pradhan Mantri Fasal Bima Yojana (PMFBY), aiming to increase the insurance coverage to as much as half of the cropped area by providing large subsidies on the cost of insurance (farmers will only pay two per cent of the sum insured for *kharif* crops such as rice, with the rest of the cost provided as government subsidies). The structure of insurance pricing under PMFBY – with a variable and unlimited subsidy amount – seems particularly unsustainable, and arguably, there are other public investments that could be made to support agriculture and rural development without distorting insurance markets.

This paper provides some of the first evidence on the demand for a bundled risk management product consisting of a drought-tolerant rice cultivar and a specially calibrated index insurance product. As such, this study provides important evidence about a potential alternative risk management product that may be viable without massive government subsidies such as those offered under PMFBY.

In the first year of our study, with modest discounts being offered, more than half of the participants in each of two treatment arms purchased some form of risk management, with 63 per cent of treated farmers purchasing the DT seed and nearly 59 per cent of treated farmers purchasing the bundled DT-WII product. Take up rates for both products fell during the second year, with 45.3 per cent uptake of the DT cultivar and 35.7 per cent uptake of DTWII product. Given the low levels of WII uptake recorded in the literature, even in the presence of discounts and subsidies, the uptake of DT-WII without financial incentives is relatively high.

Furthermore, the decline in uptake from the first year to the second year cannot be explained by the removal of product discounts. Consequently, although farmers may be sensitive to the price for risk management coverage, framing a price reduction as a subsidy or discount does not influence farmer demand. This is a very important result and has significant implications for agricultural insurance policies in India. Recall the nature of the existing agricultural insurance program, PMFBY. Under this program, farmers only pay a small amount of the sum insured, with the remainder being covered by government subsidies. While this would have the effect of significantly lowering the cost that farmers have to pay for insurance, which would likely result in increased insurance uptake, it does so at a considerable expense to the government. Our results here suggest that these public funds might be more effectively allocated towards investments that can lower the cost of risk management in a more sustainable fashion and without distorting insurance markets. Examples of more sustainable investments might include investing in crop research and development (e.g. to generate varieties that exhibit even greater drought tolerance), expanding the coverage of weather stations, or increasing the utilisation of remote sensing or satellite images for weather indices. Developing better DT varieties could result in lower-cost complementary insurance products, as reduced crop losses would need to be insured against for a particular drought severity. By improving the coverage of weather stations or using lower-cost technologies, policymakers can improve the design of the complementary index insurance products, reduce the administrative costs, and reduce basis risk, all of which would either reduce the actuarially fair cost of insurance, reduce the administrative loads that would need to be charged, or change the perceived benefit–cost ratio in favour of increased uptake.

Our results also suggest an important role for learning from experiences, as experiences from the first year of the experiment seem to have a significant impact on purchase decisions during the second year of the experiment. Importantly, however, the effect of learning seems to differ somewhat across the two products. In the case of DT, there is evidence of learning both from one’s own experiences and the experiences of others, especially if they experienced a drought (or, at least, if a drought was recorded at the block weather station) during the first year. In the case of DT-WII, having purchased the product in 2015 and experiencing a drought has a positive effect on purchasing decisions in 2016, as farmers begin to appreciate the benefits of the bundled product. But they do not learn much from the experiences of their peers, however, as peer’s experiences with the DT-WII product in 2015 have no effect on farmers’ purchasing decisions in 2016. We attribute this to product complexity more than anything else. While it is easy to look out into someone’s field and observe the performance of a particular variety vis-á-vis another variety, it is not easy to observe one’s experiences with insurance, particularly a small-scale insurance program like the one introduced here.

Another significant takeaway from this paper is that social networks matter during product marketing. The one factor that is almost ubiquitous in explaining demand, for both products across both years, is within-village peer effects – farmers’ purchase decisions are highly correlated with the purchase decisions of other farmers in their village. While we cannot truly identify the nature of this effect, it may prove beneficial for public or private sector efforts to increase insurance coverage across India.

Finally, this study points to the need for ongoing assessments of development initiatives. While the nature of the intervention did not change from 1 year to the next, we did find evidence that the factors affecting demand changed somewhat from 1 year to the next, suggesting that there is much to be gained by studying demand over multiple time periods, particularly if the product in question is relatively novel or complex. One would hope that over time the demand determinants would converge to some steady state, but in early years, it may be difficult to draw generalisable inferences.

## Data Availability

The data that support the findings of this study are available on request from the corresponding author. The data are not publicly available due to privacy or ethical restrictions.

## Footnotes

1 Farmers’ insurance uptake varies considerably by state. According to 2013 data from the Agricultural Insurance Company of India (AIC), nearly 30 per cent of farmers in Odisha held crop insurance covering *kharif* season crops, compared to just 8 per cent of farmers in Bihar and 4 per cent in Uttar Pradesh.

2 Examples of secondary traits that were considered include duration (e.g., developing short-duration varieties which have proven effective for escaping terminal drought stresses), root architecture (e.g., root depth and ability to penetrate hard soils), leaf water potential (e.g., leaf rolling, which reduces water loss in addition to reducing leaf area exposed to heat and light radiation), panicle water potential, osmotic adjustment and relative water content (Fukai *et al.,* 1999; Price and Cutrois 1999; Liu *et al.,* 2007; Kumar *et al.,* 2008; Serraj *et al.,* 2009).

3 Even dry spells of a relatively short duration can result in substantial yield losses if the dry spell occurs around the crops’ flowering stage (Serraj *et al.* 2009).

4 The concept of crop insurance goes back even further. After observing a severe storm in the French countryside in 1788, Benjamin Franklin observed ‘I have sometimes thought that it might be well to establish an office of insurance for farmers against the damage that may occur to them by storms, blight, insects, etc. A small sum paid by a number of farms would repair such losses and prevent much distress’ (Franklin 1788).

5 Moral hazard is sometimes referred to as the ‘hidden action’ problem, because it entails farmers altering their production practices (unbeknownst to the insurer) in such a way that it increases the their likelihood of receiving a payout. Adverse selection is sometimes referred to as the ‘hidden information’ problem, because the potential insured have more information about their production risks and are thus better able to assess the actuarial fairness of their insurance costs, thus resulting in the most risky producers being the most likely to want insurance (Miranda and Farrin 2012).

6 The term ‘basis risk’ is derived from the derivatives literature and is used to refer to any form of contract nonperformance. In the insurance literature, it is similar to form of contract nonperformance studied by Doherty & Schlesinger (1990), namely default risk. But with weather index insurance, there is the added possibility of upside contract nonperformance in which the policy pays out when there are no losses experienced on the farm (Clarke 2016).

7 At the time this study began in 2015, Bant and Agarpada were two separate blocks. Soon after the project began, they were merged into one block. For the purpose of this study, however, we treat them as two separate blocks.

8 Villages with fewer than 100 households were excluded from consideration prior to village selection, as such villages were deemed to be too small for the purpose of our study.

9 We incorporated two inclusion criteria to determine eligibility for participation. First, households had to cultivate rice as their primary crop. Second, they had to own at least some of the land they were cultivating. Due to the prevalence of rice cultivation in Odisha and widespread land ownership (although small in size), few households were ultimately excluded from participation. There were no requirements in terms of landholding size, so the resulting sample resulted in a fairly wide socioeconomic distribution.

10 One village that was originally allocated to the DT group was accidentally left out of the marketing sessions.

11 While it may seem intuitive that an additional treatment group offered only a WII product would be useful, we note that a stand-alone WII product meant to provide similar levels of risk management would be considerably different (and more expensive) than the specially calibrated WII product bundled with the DT cultivar, so would have generated little in terms of valid comparative analysis against the other treatments.

12 In reality, the 30 per cent premium is considerably lower than what insurance companies would likely add, but since this product was designed for a pilot program with a population that has had very little experience with insurance, we were reluctant to charge more than this modest premium.

13 Recall the earlier assumption (drawn from published estimates in Verulkar *et al.,* 2010) that *Sahbhagi dhan* yields decline by 38 and 69 per cent under moderate and severe drought stress, respectively. These yield reductions are considerably less than the yield reductions for Swarna, the most commonly grown variety in our sample area (58 and 89 per cent under moderate and severe drought stress, respectively). Assuming that the two varieties produce a homogeneous output that can be sold at the same market price, this implies that cultivating *Sahbhagi dhan* results in smaller overall crop production losses that would need to be insured. Since the potential insured losses are less, the cost of insuring production losses is also less. This illustrates how the DT component reduces the cost of drought insurance, amounting to roughly INR 50 in Balasore, INR 56 in Bhadrak, and INR 35 in Mayurbhanj (32–33 per cent of the actuarially fair cost of insuring farm income losses from drought). This does not even consider the fact that bundling the drought-tolerant seed with WII also reduces the insurer’s value-at-risk, which has significant implications for the cost of reinsurance, much of which might also be passed on to the insured in the form of higher administrative loads on top of the actuarially fair cost.

14 The recommended seeding rate for *Sahbhagi dhan* assumes transplanted rice cultivation, as is commonly practiced in the study area; cultivation by broadcasting or direct seeding methods would require larger quantities of seed; and study participants were provided information on the appropriate seeding rates for these alternative methods.

15 See Attanasio and Kaufmann (2008) for discussion of the stepwise uniform distribution in the calculation of subjective beliefs distributions.

16 While participants were informed that the index would be based on such localised rainfall measurements, they were not explicitly informed of where the rain gauges were to be installed, thus eliminating an important source of moral hazard that could imperil the insurance product.

17 The grouped nature of our sampling frame suggests that error terms should be correlated within village clusters, thus introducing a very specific form of heteroskedasticity that would bias parameter estimates in the maximum likelihood estimation of an econometric model that is nonlinear in the parameters (like a logit or a probit; see Greene 2003, pp. 673–674). Furthermore, although our data are experimental in nature, potential endogeneity (arising from, among other potential sources, omitted variables) precludes clean identification in our first stage regression. For these reasons, we report the first part estimates from a linear probability model with robust standard errors, rather than a logit or probit model. Though not reported here, we note that estimating probits for the first step yields similar results.

18 Note that $y_{-i,1}^{j}$ is constructed as the sum of all other market participants apart from individual *i*, divided by *n* − 1, where *n* is the number of study participants in the village.

19 In the present context, we permit all characteristics that condition the participation decision to also condition the consumption decision.

20 For example, in the context of health insurance program in India, Ito and Kono (2010) argue that this phenomenon reflects individuals’ use of insurance as a commitment device that facilitates ‘prepayment’ of healthcare expenses, relying upon the assumption that individuals are aware of their own self-control failures and inability to save to buffer against future expenses.
