# Supplementary material for: Drought-tolerant rice, weather index insurance, and comprehensive risk management for smallholders: evidence from a multi-year field experiment in India
Source: Aust J Agric Resour Econ. 2019 Oct 15;59:1–34. doi: 10.1111/1467-8489.12342 (PMC7188305; doi:10.1111/1467-8489.12342)
Supplement: Supplementary file 1 [file ARE-2019-1467-8489-12342-s1.pdf]

A Design of complementary weather index insurance

To determine the insurance payments that would be made under moderate and severe drought stress, we first estimated the value of lost output that farmers in these three districts might expect under moderate, severe, and extreme drought stress levels. Using historical data on district-level rice yields, we estimated a simple linear regression of yields against a time trend that smoothes out annual fluctuations due to weather variability. The results of this simple linear regression were then used to predict the expected yields under more or less optimal conditions for *kharif* 2015 and 2016. We relied upon published estimates (Verulkar et al., 2010) and assumed that yield losses under moderate and severe drought stress conditions would be 38 and 69 percent for *Sahbhagi dhan*. Next, we used historical data on the minimum support price (MSP) for rice in India and extrapolated forward to generate an estimate for the price per kilogram of rice production. Since the farm gate price is often considerably less than the MSP (e.g., due to transportation costs, transaction costs, or aggregators with market power offering prices below the MSP), we estimated the value of a kilogram of rice at the farm gate to be 20 percent less than the MSP. With this price in mind, we arrived at an estimate for the value of lost output under moderate and severe drought stress conditions.

Pricing index insurance requires consideration of the probability that index strike points will be realized and the corresponding payments that will be made if such strike points are reached, as well as any additional administrative loadings required by the insurer. The two strike points for our rainfall-based WII product were "moderate" and "severe" droughts, both of which warrant further explanation. While a drought could simply be a deviation in cumulative rainfall over the course of the entire season relative to long-term averages, the most obvious form of drought in our monsoon-season context may be the occurrence of a prolonged dry spell during the course of the season, particularly during key periods of the season associated with crop emergence, establishment, and growth. Because prolonged dry spells can be classified as extreme weather events, it is appropriate to model these extrema using an extreme value distribution. The generalized extreme value (GEV)

distribution function takes the form

$$F(x;\xi,\alpha,\kappa) = \exp\left\{-\left[1+\kappa\left(\frac{x-\xi}{\alpha}\right)\right]^{-1/\kappa}\right\} \tag{5}$$

where x is a datum on an extreme event (i.e., the length of a dry spell), $\xi \in \mathbb{R}$ is the distribution location parameter, $\alpha > 0$ is the distribution scale parameter, and $\kappa \in \mathbb{R}$ is the distribution shape parameter. These parameters can be estimated using maximum likelihood, and the estimates can be used to determine return levels, return periods, and the probability of an extreme event occurring. If the set $\{x_i\}$ is independent and identically distributed from a GEV distribution, then the log-likelihood function for a sample of n observations $\{x_1, x_2, ..., x_n\}$ is

$$\ln\left[L\left(\xi,\alpha,\kappa|x\right)\right] = \sum_{i=1}^{n}\left\{-\ln\alpha - \left(1+\frac{1}{\kappa}\right)\ln\left[1+\kappa\left(\frac{x_i-\xi}{\alpha}\right)\right] - \left[1+\kappa\left(\frac{x_i-\xi}{\alpha}\right)\right]^{-1/\kappa}\right\} \tag{6}$$

Using this log-likelihood function, we use daily rainfall data from each of the three focal districts from 1940 to 2011 to obtain estimates for the district-specific location, scale, and shape parameters characterizing the distribution of these maxima. With estimates $\hat{\xi}$, $\hat{\alpha}$, and $\hat{\kappa}$ we can then estimate either the probability p of a specific event x_p occurring, or determine what event x_p will occur with specified probability p. Based on figures reported in Kar et al. (2004), the probability of a moderate drought in Balasore and Mayurbhanj is 26.67 percent and 20.94 percent, respectively, while the probability of a severe drought in Balasore and Mayurbhanj is 2.23 percent and 2.33 percent, respectively.[21] We can then estimate the length of a dry spell corresponding to these different drought stress levels in each of the three districts:

$$x(p) = \hat{\xi} - \left(\frac{\hat{\alpha}}{\hat{\kappa}}\right)\left\{1 - \left[-\ln\left(1-p\right)^{-\hat{\kappa}}\right]\right\}$$

Actuarially fair insurance is priced such that the cost of insurance equals the expected payout received. Consider a simple index insurance product with discrete strike points, $i = 1, ..., n$, and

[21] Bhadrak district was carved out of Balasore district in 1993. In light of this fact, and due to the similarities in agroecological conditions between Bhadrak and Balasore, the drought probabilities for Balasore double as the drought probabilities used in pricing the insurance component in Bhadrak.

let p_i define the probability of an event triggering strike point i occurring. Let I_i be the insurance payout under strike point i. Then an actuarially fair cost of insurance would be $A = \sum_{i=1}^{n} p_i I_i$.

Of course, since the actuarially fair cost of insurance reflects an insurer's expected payouts, in the long run insurers should not expect to earn any economic profits, resulting in an unsustainable business model. Furthermore, since weather risk is primarily a covariate risk, insurers generally must be compensated for the risks they are exposed to in insuring many people who all share that risk. To address both of these concerns, insurers almost always incorporate a sizable premium on to the actuarially fair cost of the insurance to cover both risk and administrative burdens.

References

Kar, G., James, B., Singh, R. and Mahapatra, I.C. (2004). Bulletin 22/2004. Agro-Climate and Extreme Weather Analysis for Successful Crop Production in Orissa. Water Technology Centre for Eastern Region, Bhubaneswar, Orissa.
