# Supplementary material for: Drought-tolerant rice, weather index insurance, and comprehensive risk management for smallholders: evidence from a multi-year field experiment in India
Source: Aust J Agric Resour Econ. 2019 Oct 15;59:1–34. doi: 10.1111/1467-8489.12342 (PMC7188305; doi:10.1111/1467-8489.12342)
Supplement: Supplementary file 2 [file ARE-2019-1467-8489-12342-s2.pdf]

B Insurance and basis risk experiential learning module

Instructions: In this section you will be guiding the respondents through an experiential learning exercise where the respondents will be learning about concepts of weather index insurance and basis risk through a series of questions and experiences in drawing different colored balls out of a bag. Since understanding risks requires some basic understanding of randomness and probability, it is important that these experiences help the respondents garner a rudimentary understanding of these sometimes complex and abstract concepts. Through these learning experiences, respondents will ideally be well informed to make choices about whether to purchase risk management. This scripts have been specifically created to maximize the learning potential, so it is very important that you read through the script word-for-word. When there are directions for you (not to be read aloud to the respondent), these will be indicated by square brackets: [...]. Read through these instructions and questions slowly so that the respondent understands. Do not try to guide or influence the respondents in any way.

Q. What would you do if you experienced a moderate or severe drought and were to face serious income/crop losses, and needed to find some money to feed your family or meet other family obligations?

[**Enumerator:** There are many possible answers to this question. Some of the coping mechanisms used by respondents include the following alternatives:

- Using personal savings
- Selling assets
- Cut down on food consumption
- Selling crop inventory to meet household obligations
- Relying on others? relatives, neighbors, social organizations, such as religious group
- Using financial instruments like credit.]

Q. Have you ever heard of insurance as a way of managing drought risk?

Enumerator: Drought index insurance is one way that farmers can deal with drought risk. With this insurance, farmers pay a small amount now, and receive a cash payment at the end of the season if there has been a drought that occurred during the *kharif* season.

We determine whether a drought has occurred based upon rainfall measurements collected at weather stations throughout Khaira and Oupada blocks in Balasore; Agarpada and Bant blocks in Bhadrak; and Kaptipada and Thakurmunda blocks in Mayurbhanj. At these weather stations, we will collect information on the amount of rainfall that falls during the *kharif* season. If a drought is recorded at these stations, the insurance will provide a payment at the end of the season. In your case, the weather measurements will be based off of the weather station in your block.

The amount of the cash payment depends upon the severity of the drought, but these payments are meant to reflect the average losses in farm income from these types of droughts on a 10 decimal (0.1 acre) plot of land. For example, during a moderate drought, farm income losses for a farmer growing Swarna on a 10 decimal plot of land might be approximately INR _____. Therefore, if the farmer purchased insurance to protect against these losses in farm income, then the payment would be INR _____ if there was a moderate drought. Similarly, during a severe drought, farm income losses for the same farmer growing Swarna on a 10 decimal plot of land might be approximately INR _____. If the farmer purchased insurance to protect against these losses in farm income, then the payment would be INR _____ if there was a severe drought.

If the farmer does receive a payment from the insurance, there are no rules about how this payment is to be spent. Payments can be used

- To offset costs of additional irrigation to supplement deficient rainfall
- For purchasing seed, fertilizer, or land rent for future cultivation
- For financing food purchases for the household
- For paying school fees, medical costs, clothing, etc.
- For investments, like purchasing oxen, cows, donkeys, etc.

Q. Are there any restrictions about what insurance payments can be used for?

[**Enumerator:** Ensure that the respondent understands that there are no restrictions on how the insurance payments can be spent.]

Q. Are the weather conditions at your farm always the same as the weather conditions at the block headquarters?

[**Enumerator:** Ensure that the respondent understands that weather conditions vary from one location to the next.]

As we have mentioned, the insurance will pay out if there is a drought measured at the weather station that is located in your block. Because your farm may be located far away from the weather station, there is a chance that the weather conditions on your farm may not be the same as the weather conditions at the weather station. Recall that the insurance depends upon the observed rainfall at the weather station, not rainfall on your field.

To illustrate this scenario, consider this bag, which has 1 pink ball and 1 green ball. In just a moment, I will ask you to draw a ball out of this bag. If you draw a pink ball, then the weather conditions on your farm will be the same as the weather conditions at the local weather station. If you draw a green ball, then the weather conditions on your farm will be different from the weather conditions at the local weather station. This is meant to reflect the fact that your conditions may not be the same as the weather conditions at the weather station.

It is possible that you may experience poor weather conditions on your field, but the weather conditions may be fine at the weather station. This is illustrated by the green ball, which indicates that the weather conditions do not match. Since the insurance contract is based on the rainfall measurements at the local weather station, and not your actual farm income losses or the actual rainfall you experience on your farm, there is a chance that the insurance contract would not compensate you for farm income losses even if you experience a loss in farm income resulting from a drought.

[**Enumerator:** Show the respondent Figure **??**. Ensure that the respondent understands that he/she may experience drought conditions on his/her farm, but that there might not be drought conditions at the closest weather station.]

Alternatively, it may be the case that weather conditions on your farm are very similar to the

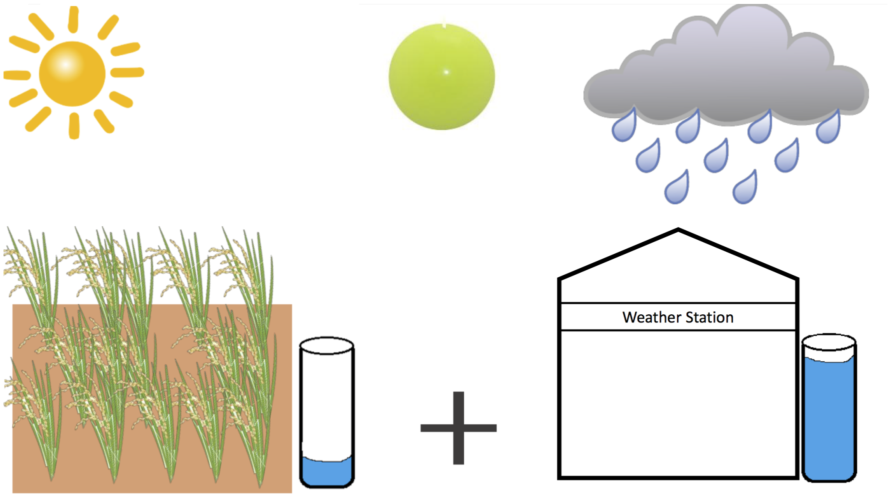

Figure 4. Example of mismatch between weather conditions on farmer's field and weather station where index measurements are recorded

weather conditions at the weather station. If there is a drought on your farm, there is also a drought at the weather station. Under this situation, you would receive a payout from the insurance policy. Because the weather conditions on your farm are similar to the weather conditions at the local weather station, this would be like the situation of drawing a pink ball from the bag.

[**Enumerator:** Show the respondent Figure **??**.]

Q. Do you understand how the insurance payouts operate? Are the payouts based on income losses on your farm? Or are the payouts based upon observed rainfall at a weather station?

[**Enumerator:** Ensure that the respondent understands that the payouts are not based on income losses on their farm, but are based upon measured weather at a local weather station in his/her block.]

Now, imagine that you have experienced a drought on your farm. There was little rainfall during the year, and your paddy crop suffered. If you had purchased insurance, you may be entitled to a payment from the insurance contract if there was a drought detected at the local weather station. But the weather conditions at the local weather station might not be the same as the weather conditions on your field. If the weather conditions are different, you will not receive a payment from the insurance, since these payments are based on the weather conditions at the local weather station and do not depend upon the actual weather conditions on your farm.

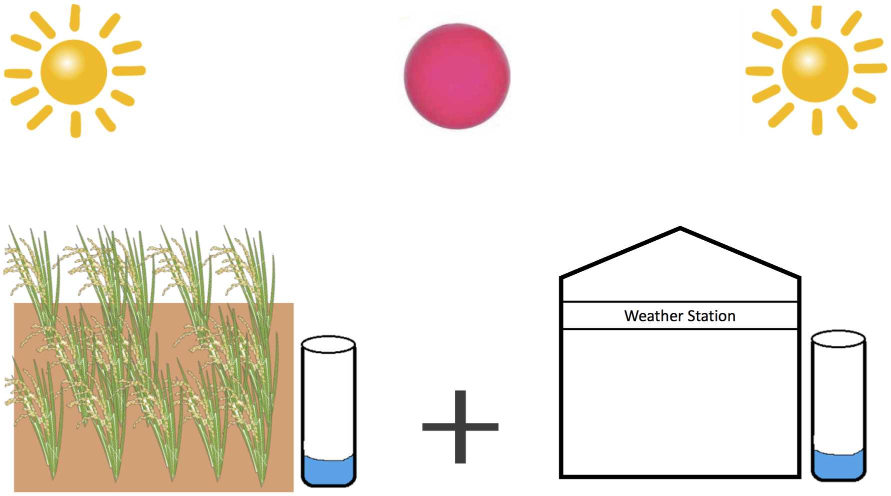

Figure 5. Example of match between weather conditions on farmer's field and weather station where index measurements are recorded

Please draw a ball from this bag to see whether or not the weather conditions on your farm were the same as the weather conditions at the local weather station.

[**Enumerator:** Please have the respondent draw a ball from the bag]

Q. What color is the ball?

Q. If you had insurance and experienced farm income losses from a drought that occurred on your farm, what would drawing this color ball imply for receiving a payout?

[**Enumerator:** Ensure that the respondent understands that, if they drew a pink ball, that the weather conditions on their farm would be the same as the weather conditions at the local weather station, so they would receive an insurance payment. If, however, they drew a green ball, this indicates that the weather conditions on their farm are different than the weather conditions at the local weather station, and they would not receive an insurance payout.]
