# Supplementary material for: Drought-tolerant rice, weather index insurance, and comprehensive risk management for smallholders: evidence from a multi-year field experiment in India
Source: Aust J Agric Resour Econ. 2019 Oct 15;59:1–34. doi: 10.1111/1467-8489.12342 (PMC7188305; doi:10.1111/1467-8489.12342)
Supplement: Supplementary file 3 [file ARE-2019-1467-8489-12342-s3.pdf]

C Drought risk experiential learning module

Instructions: In this section you will be guiding the respondents through an experiential learning exercise where the respondents will be learning about the concept of drought risk through a series of questions and experiences in drawing different colored balls out of a bag. Since understanding risks requires some basic understanding of randomness and probability, it is important that these experiences help the respondents garner a rudimentary understanding of these sometimes complex and abstract concepts. Through these learning experiences, respondents will ideally be well informed to make choices about whether to purchase risk management. This scripts have been specifically created to maximize the learning potential, so it is very important that you read through the script word-for-word. When there are directions for you (not to be read aloud to the respondent), these will be indicated by square brackets: [...]. Read through these instructions and questions slowly so that the respondent understands. Do not try to guide or influence the respondents in any way.

Enumerator: As a farmer, you face many different types of risks related to your paddy production.

Q. In your opinion, what are some of the most serious risks to paddy production during the *kharif* season?

[**Enumerator:** Some of the possible responses include

- Pests, weeds, diseases
- Crops being eaten or trampled by animals
- Lack of seed/fertilizer
- Soil type
- Frost/wind
- Lack of rainfall (drought)
- Excess rainfall (flooding)
- Sickness in the household, death, etc. (influencing labor supply)

You may need to guide or nudge the respondent in the identification of these risks. Ensure that the respondent acknowledges weather to be a significant constraint, particularly drought.]

Enumerator: In this exercise, we are interested in understanding your preferences towards various methods for managing risks related to paddy production – particularly those related to droughts during the *kharif* season. There are many instances in which droughts can occur during the *kharif* season, including late monsoon arrival or early monsoon cessation, or prolonged periods without rainfall, or simply just below average rainfall. The India Meteorological Department (IMD) defines a moderate drought as any occurrence in which rainfall is between 26-50 percent below normal, while a severe drought is any occurrence in which rainfall is more than 50 percent below normal. The impact of the drought on paddy production depends on several key factors, including the paddy variety that you cultivate, the timing of the drought, the severity of the drought, whether or not you have access to irrigation, your soil type, and many other potential factors. Some of these factors are directly under your control, while others are not. You do not have control over the weather, so you cannot influence the timing or severity of droughts if they occur.

Q. Do you understand how droughts can affect paddy production?

[**Enumerator:** Ensure that the respondent understands that droughts can affect their paddy production. It should be pretty obvious to respondents that droughts affect their paddy yields. They may also indicate that they delay transplanting or cultivate less land if the monsoon is delayed.]

Enumerator: Droughts are the result of weather. Weather is risky because it is difficult to predict with a great deal of certainty. To illustrate drought risk, consider this bag, which contains 5 balls: some of these balls are green, while some balls are red. In a moment, we will ask you to draw a ball from this bag. Imagine that drawing a ball from a bag is like observing weather during a given *kharif* season. If the ball is green, this is like observing good weather during the *kharif* season. If the ball is red, this is like observing a moderate drought during the *kharif* season.

[**Enumerator:** Please ask the respondent to draw a ball from the bag]

Q. What color is the ball?

Q. Does this indicate observing good weather or observing a moderate drought during *kharif*?

[**Enumerator:** depending upon the color of the ball that is chosen, make sure that the respondent understands what weather outcome is implied by his drawn ball.]

[**Enumerator:** place the ball back in the bag]

Enumerator: Now, I?m going to have you draw 5 balls from the bag, one at a time.

[**Enumerator:** Please have the respondent go through a sequence drawing 5 balls from the bag, replacing the drawn ball each time (there should always be 5 balls in the bag each time the respondent makes his/her draw).]

Q. How many green balls did you draw? How many red balls did you draw?

Q. There are 5 balls in this bag. How many do you think are green? How many do you think are red?

[**Enumerator:** After the respondent has guessed how many green and red balls he/she thinks are in the bag, take all of the balls out of the bag to show him/her the actual number of green and red balls there are.]

Since there are 4 green balls and 1 red balls, we should expect that, if we draw 5 balls in a row, we would draw 1 red ball. This is similar to observing weather over 5 years: we would expect that 1 of those years would be a moderate drought year.

I?m now going to have you draw 5 more balls from the bag.

Q. How many red balls do you expect to draw?

[**Enumerator:** make sure the respondent understands that he/she should expect to draw 1 red ball from the bag].

[**Enumerator:** Please have the respondent go through a sequence drawing 5 balls from the bag, one at a time, replacing the drawn ball each time (there should always be 5 balls in the bag when the respondent makes his/her draw).]

Q. How many green balls did you draw? How many red balls did you draw?

Q. Is this more or less than you expected (or exactly as many as expected)?

Enumerator: You can never know for sure what color ball you will draw until you actually draw it. You may expect that you will draw 1 red ball in 5 draws, but there is nothing guaranteeing that pattern. You may draw 2 red balls in a row, or you may go several draws without drawing a

red ball.

The same is true for weather.

Weather is difficult to predict. We may expect 1 moderate drought every 5 years, but we can never be sure that we will only observe 1 moderate drought in any 5 year period, or that we will observe a single drought during that period. In other words, we may observe several moderate drought years in a row, or we may observe a period of several years without a moderate drought.

So far, this we have only talked about moderate drought. We expect that we should observe a moderate drought 1 time during any 5-year period. But, as we have seen, we may experience more or fewer moderate droughts than what we expect.

Severe droughts have a greater impact than moderate droughts. They either last longer or result in a greater shortage in rainfall compared to moderate drought. But severe droughts occur less often than moderate droughts. We would not expect a severe drought to occur 1 time every 5 years. As such, if we had balls in a bag of balls representing observed weather, we would not have 1 red ball representing a severe drought and 4 green balls representing normal rainfall. If we wanted to use balls like these to represent the likelihood of a severe drought, we would need 1 red ball and 49 green balls

Q. Based on what we have discussed, if you were to observe weather over a 5-year period, during how many of those years would you expect to observe a moderate drought?

[**Enumerator:** Ensure that the respondent understands that he/she would expect to observe 1 moderate drought over a given 5-year period].

Q. Is it possible that you could experience more than 1 moderate drought during a 5-year period?

[**Enumerator:** Ensure that the respondent understands that it would indeed be possible that he/she observe more than 1 moderate drought over a 5-year period].

Q. Is it possible that you would not experience a single moderate drought during a 5-year period?

[**Enumerator:** Ensure that the respondent understands that it would indeed be possible that he/she fails to observe a single moderate drought over a 5-year period].

Q. Which is more likely to occur, a moderate drought or a severe drought?

[**Enumerator:** Ensure that the respondent understands that a moderate drought is more likely to occur than a severe drought. On average, we expect a moderate drought to occur 1 time every 5 years. We would only expect a serious drought to occur 1 time every 50 years. The actual occurrence of moderate and severe drought may be more or less frequent than what we expect].
