# Supplementary material for: Drought-tolerant rice, weather index insurance, and comprehensive risk management for smallholders: evidence from a multi-year field experiment in India
Source: Aust J Agric Resour Econ. 2019 Oct 15;59:1–34. doi: 10.1111/1467-8489.12342 (PMC7188305; doi:10.1111/1467-8489.12342)
Supplement: Supplementary file 4 [file ARE-2019-1467-8489-12342-s4.pdf]

D Impact of DT and DT-WII on rice yields

Table D1. Two-stage least squares estimates of local average treatment effect: Impact of DT and DT-WII on rice yields

	Yield impacts (2015)
Purchased DT	0.203
	(1.007)
Purchased DT-WII	-0.391
	(2.831)
Purchased DT × Drought occurred	0.073
	(0.295)
Purchased DT-WII × Drought occurred	0.729
	(3.098)
Drought occurred in 2015	-0.471
	(5.514)
R^2	0.20
N	2,161

Source: Authors. Note: Endogenous regressors are instrumented using random allocation to treatment groups and interactions between random allocation to treatment group and drought experiences. Regression contains an intercept term and controls for yields from previous season (McKenzie, 2012) and characteristics for which there were significant differences between households in the control group and households in the treatment groups (household size, land ownership, rice area, total rice output, and trust and asset indices). t-statistics based on standard errors clustered at the village level in parentheses.

References

McKenzie, D. (2012). Beyond baseline and follow-up: the case for more T in experiments, Journal of Development Economics 99, 210–221.
